# Pathology of Trench Frost-Bite

**Published:** 1918-02

**Authors:** 


***Pathology of Trench Frost-Bite.*** By LORRAIN SMITH, JAMES RITCHIE, and JAMES DAWSON, Univ. of Edin. Abs. in the *Brit. Med. Jour.*, Dec. 25, 1915, p. 933 of an article in the *Jour. of. Bact. and Path.*, vol. XX, p. 159.

The authors divide cases of frost-bite, a term which they employ for convenience, into two groups according to the nature of the lesions: those in which extreme cold directly and immediately produced death of the tissues, and those in which cold impairs the vitality of the tissues with or without subsequent death of the affected cells.

The observations recorded were partly from experiments upon animals and partly from clinical cases of soldiers invalided from the front. From these observations the authors conclude that the essential change consists in the damage to the blood vessels. There is probably an initial constriction of the vessels; when this passes off the swelling of the feet which results from the damaged condition, occurs while the circulation is being restored to the normal. The damage is evidenced by the swelling of the endothelial cells in vessels of all kinds, and by the vacuolation of the muscle fibers in the arterial walls. An excessive amount of fluid is poured out into the tissues, and in some cases the vessels rupture and hemorrhage follows. Along with the injury to the vessels there is also an interference with the vitality of the cells of the surrounding connective tissues. Evidence of this was found in the readiness with which fibrin formation occurred in the exuded fluid as contrasted with the absence of thrombi in the blood vessels. Further evidence of injury was afforded by the infiltration of the parts with leucocytes and other phagocytic cells whose function is the removal of the products of tissue destruction. These cells were abundant immediately around the blood vessels, and were scattered through the tissue spaces. The bundles of the fibrous connective tissues were separated by the accumulated fluid, and became swollen and disintegrated. It is considered probable that the etiological factors concerned in the damaging of the vessels and tissues are complex. The condition may be due on the one hand to the direct effect of the cold, and on the other to the starvation of the parts resulting from the vascular constriction and the sluggishness of the circulation generally. The necrobiotic changes affected the muscle, but the nerves were resistent, although edema was found in the axis cylinders, and was thought to indicate a slight depression of vitality. The exudation must throw a great strain upon the lymphatic drainage of the parts. Not only are the lymph channels distended with a fluid richer in proteins than natural (as shown by the presence of fibrin), but the lymph glands are called into action by the necessity of disposing of the dying cells, of the cellular products, and of the fibrinous exudates which are passing through them.

When the experimental results of moderate wet cold for long periods and the results of very low temperature for short periods were compared, it was seen that there was no difference except in degree. When the results in animals were compared with those in soldiers, a very close similarity was observed. Though the pathological changes in man could not be observed histologically, it seemed clear that a close parallelism existed between them and the artificial lesions in animals. All the clinical observations could be interpreted in the light of the experimental results. The delay in recovery is probably due to the length of time required to restore the vessel walls so as to support the strain of the changes in circulation which take place, for example, in walking. Exudates must also be cleared from the lymphatic paths.

The experiments supported the conclusions reached by Larrey in the Napoleonic wars, that heat applied during the period of the restoration of circulation tends to favor the production of necrosis. Although the heat applied by them was well below the heat of the animal's body, its damaging effect was clearly demonstrated in edematous swelling and a considerable amount of hemorrhagic infiltration. The warmth had caused congestion in the damaged foot, and the blood vessels were unable to stand the strain. Dry warm air had less effect than warm water, but under field conditions heat is likely to be applied to limbs which still have their ordinary coverings saturated with wet mud.

The authors consider pressure and constriction chiefly responsible for the condition of frost-bite. They note that symptoms are usually most marked at the points where corns and callosities are most commonly found. They remark that puttees non only tend to interrupt circulation by compression, but that by absorbing moisture, they allow the heat of the body to escape freely, even when the foot is no longer in mud and water.

